# Study protocol of the Our Futures Vaping Trial: a cluster randomised controlled trial of a school-based eHealth intervention to prevent e-cigarette use among adolescents

**DOI:** 10.1186/s12889-023-15609-8

**Published:** 2023-04-12

**Authors:** Lauren A. Gardner, Amy-Leigh Rowe, Emily Stockings, Katrina E. Champion, Leanne Hides, Nyanda McBride, Steve Allsop, Siobhan O’Dean, Matthew Sunderland, Yong Yi Lee, Cathy Mihalopoulos, Becky Freeman, Janni Leung, Hayden McRobbie, Lexine Stapinski, Nicole Lee, Louise Thornton, Jennifer Debenham, Maree Teesson, Nicola C. Newton

**Affiliations:** 1 The Matilda Centre for Research in Mental Health and Substance Use, The University of Sydney, Sydney, NSW Australia; 2 School of Psychology, The University of Queensland, Brisbane, QLD Australia; 3 National Drug and Research Institute, EnAble Institute, Curtin University, Perth, WA Australia; 4 Health Economics Group, School of Public Health and Preventive Medicine, Monash University, Melbourne, VIC Australia; 5 School of Public Health, The University of Queensland, Brisbane, QLD Australia; 6 Queensland Centre for Mental Health Research, Brisbane, QLD Australia; 7 School of Public Health, The University of Sydney, Sydney, NSW Australia; 8 National Centre for Youth Substance Use Research, The University of Queensland, Brisbane, QLD Australia; 9 National Drug and Alcohol Research Centre, University of New South Wales, Sydney, Australia; 10 National Drug and Research Institute, Curtin University, Perth, WA Australia

**Keywords:** E-cigarettes, Vaping, Tobacco, Adolescence, Prevention, eHealth, School-based intervention

## Abstract

**Background:**

Effective and scalable prevention approaches are urgently needed to address the rapidly increasing rates of e-cigarette use among adolescents. School-based eHealth interventions can be an efficient, effective, and economical approach, yet there are none targeting e-cigarettes within Australia. This paper describes the protocol of the OurFutures Vaping Trial which aims to evaluate the efficacy and cost-effectiveness of the first school-based eHealth intervention targeting e-cigarettes in Australia.

**Methods:**

A two-arm cluster randomised controlled trial will be conducted among Year 7 and 8 students (aged 12–14 years) in 42 secondary schools across New South Wales, Western Australia and Queensland, Australia. Using stratified block randomisation, schools will be assigned to either the *OurFutures Vaping Program* intervention group or an active control group (health education as usual). The intervention consists of four web-based cartoon lessons and accompanying activities delivered during health education over a four-week period. Whilst primarily focused on e-cigarette use, the program simultaneously addresses tobacco cigarette use. Students will complete online self-report surveys at baseline, post-intervention, 6-, 12-, 24-, and 36-months after baseline. The primary outcome is the uptake of e-cigarette use at 12-month follow-up. Secondary outcomes include the uptake of tobacco smoking, frequency/quantity of e-cigarettes use and tobacco smoking, intentions to use e-cigarettes/tobacco cigarettes, knowledge about e-cigarettes/tobacco cigarettes, motives and attitudes relating to e-cigarettes, self-efficacy to resist peer pressure and refuse e-cigarettes, mental health, quality of life, and resource utilisation. Generalized mixed effects regression will investigate whether receiving the intervention reduces the likelihood of primary and secondary outcomes. Cost-effectiveness and the effect on primary and secondary outcomes will also be examined over the longer-term.

**Discussion:**

If effective, the intervention will be readily accessible to schools via the *OurFutures* platform and has the potential to make substantial health and economic impact. Without such intervention, young Australians will be the first generation to use nicotine at higher rates than previous generations, thereby undoing decades of effective tobacco control.

**Trial registration:**

The trial has been prospectively registered with the Australian and New Zealand Clinical Trials Registry (ACTRN12623000022662; date registered: 10/01/2023).

**Supplementary Information:**

The online version contains supplementary material available at 10.1186/s12889-023-15609-8.

Despite only emerging in recent years, e-cigarette use, particularly among adolescents who do not smoke, has become a global public health concern [1]. The most recent (2017) representative survey of Australian secondary school students, aged 12–17 years, found 14% had used an e-cigarette, among whom 32% had done so in the past month [2]. Global data suggests these numbers are rapidly climbing, with over 40% of young people in other high-income countries (e.g., the United States [US], France, Italy and Spain) now having used e-cigarettes [3]. This dramatic increase contrasts with the steady declines in youth alcohol and other drug use observed over the past decade [4]. The surge has been fuelled by e-cigarette companies and marketing campaigns targeting youth through the use of bright colours, flavours (e.g., fairy floss), and a strong social media presence. This is problematic as e-cigarettes often contain harmful chemicals and the long-term effects are currently unknown [5]. Although, in Australia, it is illegal to buy or sell e-cigarettes containing nicotine without a prescription [6], they are surprisingly easy to purchase unlawfully from tobacconists, vape stores and online. Further, many e-cigarettes contain high doses of nicotine (up to 50 mg – equivalent to approximately one pack of cigarettes [7]), even when labelled as nicotine free [8]. Nicotine is highly addictive and can impede healthy brain development [9]. Indeed, more than half of adolescent e-cigarette users experience symptoms of nicotine dependence [10].

A major recent review found e-cigarettes can also cause a range of acute health problems, including e-cigarette or vaping use-associated lung injury (EVALI), seizures, poisoning and burns [11]. E-cigarette use has also been associated with mental health problems, such as depression and suicidal ideation in adolescence (although causality has not been established) [12]. Alarmingly, young people who use e-cigarettes are three times more likely to take up tobacco smoking when compared to people who have never used e-cigarettes, putting them at risk of the substantial harm and burden of disease that tobacco smoking can cause [13]. Whilst e-cigarettes may help some individuals to quit smoking, the health risks significantly outweigh any benefits [11]. Despite this knowledge, perceived harm of e-cigarettes by adolescents is low [14], and they are now the most commonly used nicotine product among youth [15]. Effective and scalable interventions are urgently needed to address this critical health issue which has the potential to undo decades of successful tobacco control in Australia.

Drug harm prevention initiatives delivered during adolescence are an efficient and effective way to deliver long-term health and economic benefits [16, 17]. Schools are an ideal setting as they provide an opportunity to reach large numbers of young people and intervene prior to the onset of harmful drug use [2]. In addition, drug education is mandatory within the health curriculum across many countries, including Australia [18]. School-based preventive interventions targeting tobacco, alcohol and other drugs can be effective at preventing, delaying, and reducing substance use and related harms [16, 19]. The strongest evidence exists for interventions that help adolescents overcome social influences to use tobacco or other drugs, and improve social competence, by developing problem solving, decision-making, resistance and assertiveness skills [19, 20]. Such interventions are best implemented alongside policy-level prevention initiatives, such as laws to reduce access, use and supply [21, 22].

Despite the demonstrated potential of school-based substance use prevention, an evidence-practice gap exists, with less than one in four teachers implementing a drug prevention program with evidence of effectiveness [23, 24]. Amongst schools that do deliver evidence-based prevention, many do so ‘off-label’, with teachers making adaptations over 95% of the time, undermining the fidelity and established efficacy of the programs [23, 25]. This is often due to implementation barriers, such as limited time and resources, or a lack of suitability due to end-users not being involved in program design. eHealth interventions can overcome common implementation barriers within the school environment. For example, pre-programmed content reduces reliance on teacher training, and limits the potential for adaptations that could compromise intervention fidelity. Further, online interactive components can increase student engagement, accessibility and scalability [26].

Whilst evidence supports the effectiveness of school-based preventive eHealth interventions in addressing alcohol and other drug use among adolescents [19], there is a dearth of eHealth interventions targeting e-cigarette use. Developed in the US and based on successful tobacco and other substance use prevention techniques, the ‘CATCH My Breath’ intervention is, to our knowledge, currently the only school-based eHealth intervention with demonstrated efficacy at preventing e-cigarette use [27]. However, lessons consist of teacher presentations and peer-led group work which may vary and compromise effectiveness in real-world settings. Moreover, the need for specialised teacher and peer-leader training may limit scalability. An urgent need remains for new scalable approaches to prevent e-cigarette use, specifically within the unique Australian context where it is illegal to buy or sell e-cigarettes containing nicotine [6].

To address these gaps, we developed the first school-based eHealth preventive intervention to target e-cigarette use among young Australians – the *OurFutures Vaping Program*. *OurFutures* is an innovative, universal prevention model that adopts a harm minimisation and comprehensive social influence approach to drug prevention. The model was designed to maximise intervention fidelity and overcome common implementation barriers for school-based interventions. For example, the core intervention component is the cartoon storyboards which cannot be adapted by teachers. The cartoons follow the lives of a group of young people around the same age as the target students who, through their experiences, impart knowledge, skills and values related to alcohol and other drug use. In essence, this provides peer-led education, which is more effective at addressing adolescent substance use compared to adult-led education [28]. Further, online delivery makes the program readily accessible and scalable, regardless of location. To reduce the burden on teachers, the content is aligned with the Health & Physical Education Curriculum and offers a direct replacement for regular health education lessons.

The effectiveness of the *OurFutures* programs has been established through 8 large randomised controlled trials (RCTs) across Australia (240 schools, > 21,000 students). These studies demonstrated that the *OurFutures* programs targeting alcohol, cannabis, psychostimulants and emerging drugs, are more effective than school-based health education as usual in reducing alcohol consumption, binge drinking, cannabis use, ecstasy use, harms from substance use, intentions to use substances, and in increasing knowledge about substance use harms, up to 3 years following intervention delivery [29–31]. Notably, reductions in harmful alcohol use have also been observed up to age 20 (i.e., 7 years following intervention delivery) [16]. Moreover, a recent independent review found *OurFutures* to be one of only two school-based alcohol and other drug education programs in Australia with a strong evidence base [32]. Capitalising on this world-first program of research, we have applied the successful *OurFutures* model to the prevention of e-cigarette use through the *OurFutures Vaping Program*. This study aims to evaluate the efficacy of the program through a multisite cluster RCT. It is hypothesised that compared to an active control group (health education as usual):

1. students who receive the *OurFutures Vaping Program* will be less likely to commence e-cigarette use at the 12-month follow-up (primary outcome).
2. the *OurFutures Vaping Program* will achieve superior outcomes on secondary outcomes including: uptake of tobacco smoking, frequency/quantity of e-cigarettes use and tobacco smoking, intentions to use e-cigarettes/tobacco cigarettes, knowledge about e-cigarettes/tobacco cigarettes, motives and attitudes relating to e-cigarettes, self-efficacy to resist peer pressure and refuse e-cigarettes, mental health, quality of life, and resource utilisation.
3. benefits of the *OurFutures Vaping Program* on primary and secondary outcomes will be sustained over the long-term (up to 36-months post baseline).
4. the *OurFutures Vaping Program* will demonstrate cost-effectiveness (up to 36 months post baseline).

## Method

### Study design

A two-arm cluster RCT will be conducted among Year 7 and 8 students in 42 secondary schools across New South Wales (NSW), Western Australia (WA) and Queensland (QLD), Australia, from 2023 to 2026. Cluster randomisation at the school level will be used to avoid potential contamination of the control group by the intervention group (e.g., due to student communication). This design allows for optimal and rigorous evaluation of the research questions and hypotheses. Schools will be randomly allocated (1:1) to either the *OurFutures Vaping Program* intervention group or an active control (health education as usual). An economic evaluation will be conducted alongside the cluster RCT to determine the cost-effectiveness of the OurFutures Vaping Program. The study is sponsored by the University of Sydney and has been approved by the Human Research Ethics Committees of the University of Sydney (2022/818), University of Queensland (2023/HE000082) and Curtin University (HRE2023-0059). The trial will follow Consolidated Standards of Reporting Trials (CONSORT) guidelines for cluster RCTs and has been prospectively registered with the Australian and New Zealand Clinical Trials Registry (ACTRN12623000022662), with any modifications logged immediately. This protocol follows Standard Protocol Items: Recommendations for Interventional Trials (SPIRIT) guidelines (see Additional File 1 SPIRIT checklist).

### Sample size calculations

The sample size calculations are based on a method to detect intervention by time interactions in longitudinal cluster RCTs [33]. To detect differences by state, and between groups across the six measurement occasions, six schools and 420 students will be randomly allocated to each of the control and intervention groups in each state (12 schools; 840 students per state). This will achieve 95% power to detect an Odds Ratio (OR) of 0.7 in the primary outcome, which is in line with effect sizes from similar school-based prevention trials targeting tobacco smoking [34, 35]. To account for school dropout (approx. 15%) and student attrition (approx. 25% over 36 months), we aim to recruit a minimum of 14 schools and 1,120 students per state at baseline to test intervention effects (N = 42 schools; 3,360 students). Based on rates in our previous school-based universal substance use prevention trials and successful recruitment strategies [36], we anticipate most, if not all, students will participate.

### Procedure

#### Inclusion and exclusion criteria

Eligible participants will be Year 7 and Year 8 students (aged approximately 12–14 years) attending participating schools in 2023. Students will be required to be fluent in English and provide informed active consent and parental consent to participate. Schools with less than 70 enrolled students per cohort and based outside of NSW, WA or QLD will be excluded.

#### Recruitment

Independent, Government and Catholic secondary schools in NSW, WA and QLD will be invited to participate. Schools that have previously expressed interest in participating in research will be approached and we will simultaneously promote the study through our extensive professional networks via email and social media. Schools will also be approached using publicly available contact details. An invitation will be sent to school principals and health education staff outlining the study aims and seeking permission to implement the study. Research staff will also follow up schools via phone call and email.

#### Randomisation

**Fig. 1:**
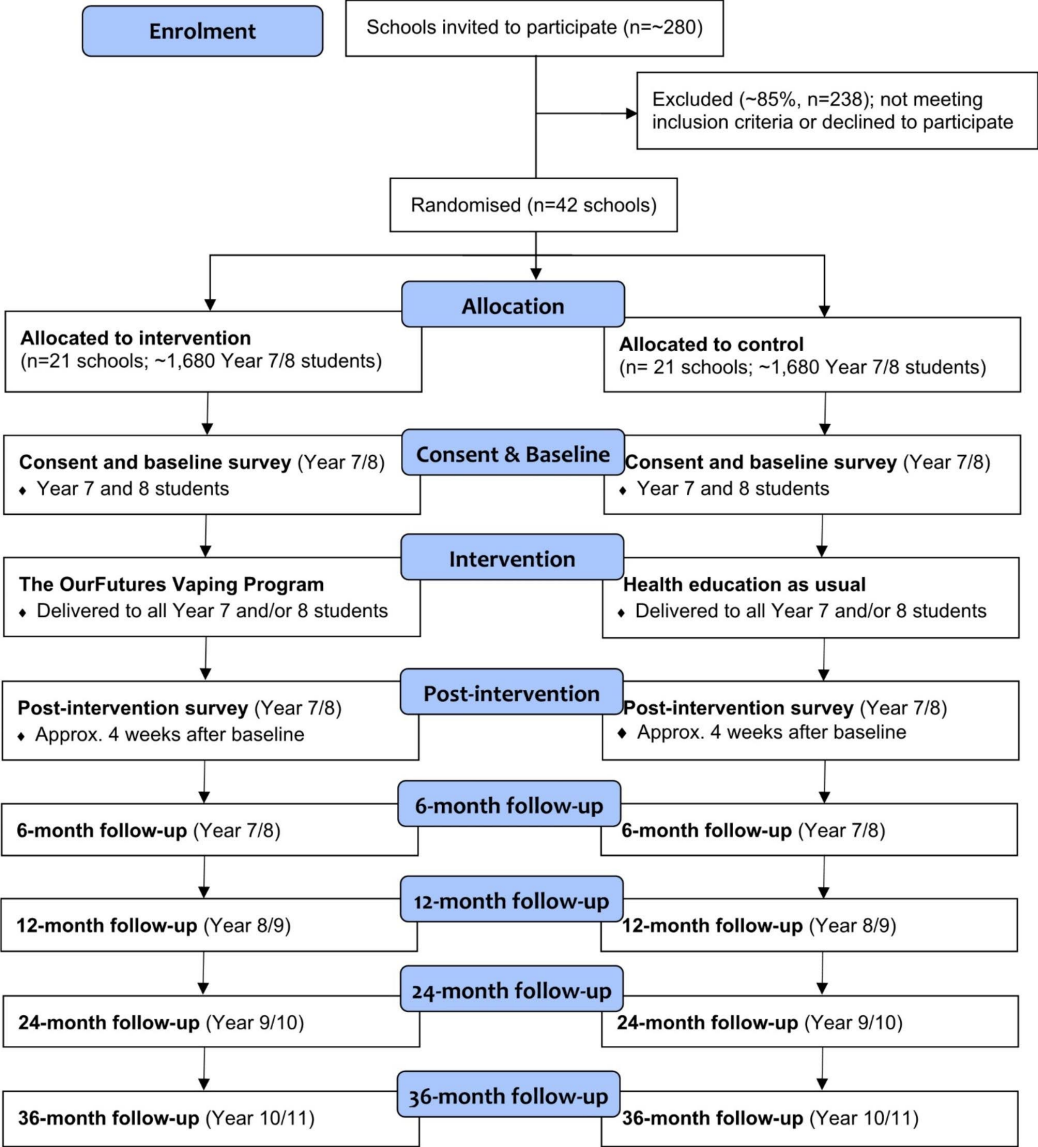
Anticipated recruitment, randomisation and assessment of participants

The *blockrand* package in R [37] will be used to generate an unpredictable, concealed random allocation sequence. After schools’ consent and enrolment in the study, a biostatistician with no role in school recruitment will use the package to block randomise schools to study groups, with stratification by state and school gender mix (coeducational, predominately male [> 60%], or predominately female [> 60%]). Automatic randomisation removes any researcher involvement, and the allocation will be concealed from the investigators and all research personnel (blinded), except those with direct school involvement where blinding is not possible (e.g., Research Assistants who will need to discuss intervention delivery with teachers). Twenty-one schools will be randomly allocated to the *OurFutures Vaping Program* intervention group and 21 schools to an active control group. Figure 1 provides an overview of the anticipated recruitment and randomisation process, and Table 1 presents the SPIRIT flow diagram schedule of enrolment, interventions, and assessments.

**Table 1:** SPIRIT flow diagram: schedule of enrolment, interventions, and assessments

	Study Period							
	Enrolment	Allocation	Post-allocation					Close-out
TIMEPOINT	-t_1_	0	t_1_ baseline	t_2_ 5 weeks	t_3_ 6 months	t_4_ 12 months	t_5_ 24 months	t_6_ 36 months
**ENROLMENT**								
Eligibility Screen	X							
School enrolment	X							
Allocation		X						
Informed consent			X					
**INTERVENTION**								
Health education as usual (active control)								
OurFutures Vaping program (intervention)								
**ASSESSMENT**								
Primary Outcomes			X	X	X	X	X	X
Secondary Outcomes (except RUQ & SDQ)			X	X	X	X	X	X
Secondary Outcomes (RUQ & SDQ)			X	X		X	X	X
Process outcomes				X				

*Note: RUQ = Resource Use Questionnaire; SDQ = Strengths & Difficulties Questionnaire.*

#### Informed consent

Participant information statements and consent forms will be distributed to parents/guardians via hardcopy or electronically (depending on school preference). Due to differing requirements across ethics committees, some schools will use passive (opt-out) parental consent, while others will use active (opt-in) parental consent. If insufficient active written parental consent is obtained from a school, verbal parental consent will be sought from the remaining parents. A school staff member will contact parents using contact details available to the school and will either be reimbursed for their time and effort, or the school will be reimbursed the costs of employing a casual teacher for a day. Students will be required to provide active written consent prior to the baseline assessment. Active written consent will also be sought from Year 7/8 teachers at both control and intervention schools to complete online evaluations and fidelity logbooks.

#### The OurFutures Vaping Program

**Fig. 2:**
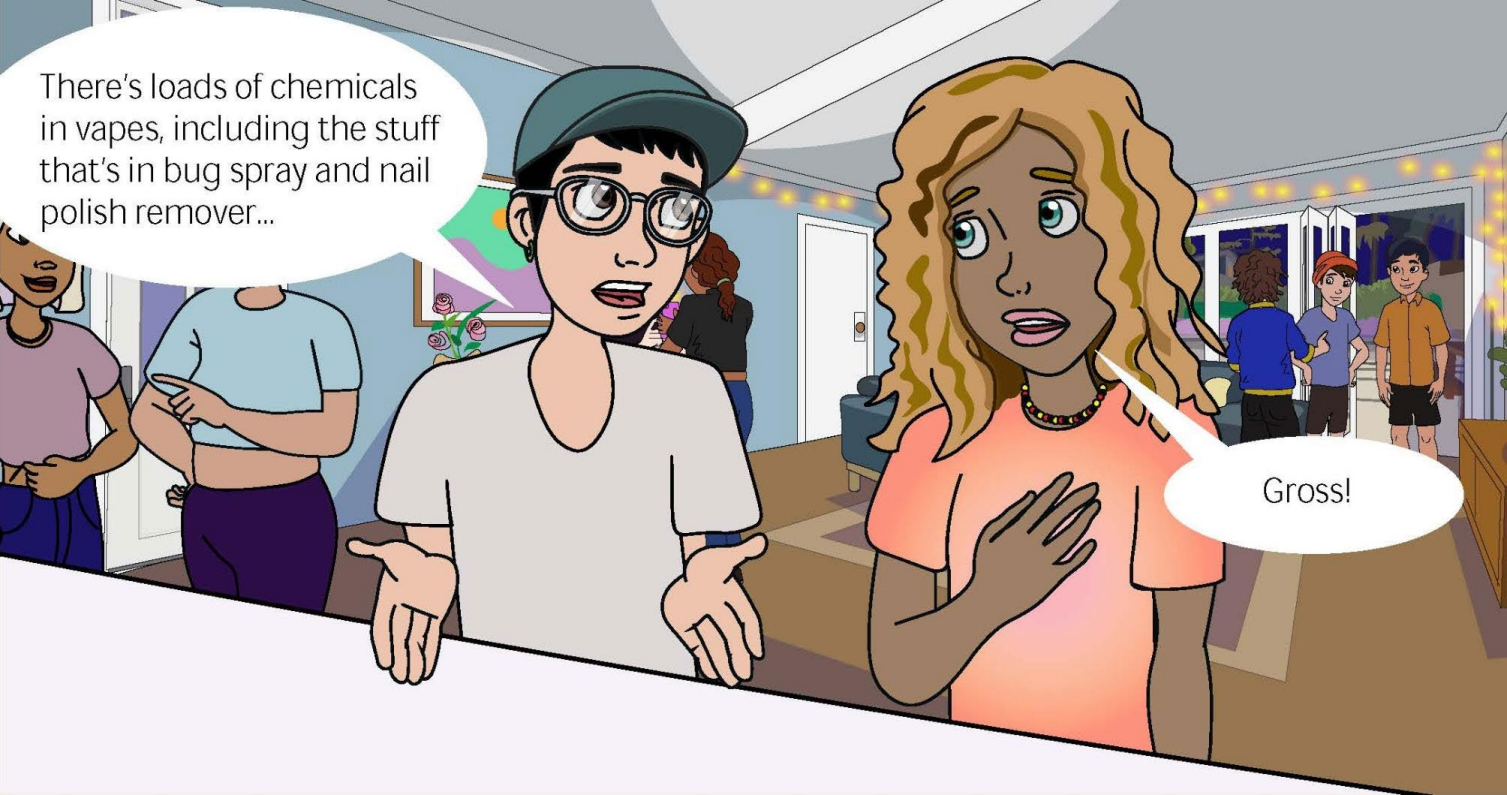
Example cartoon from the *OurFutures Vaping Program*

Schools allocated to the intervention group will implement the *OurFutures Vaping Program* in health education classes in 2023. The program involves four 40-minute lessons, ideally delivered one week apart. Each lesson consists of a web-based cartoon component (see Fig. 2) completed individually by students (approx. 20 min), followed by optional teacher-facilitated activities (e.g., quizzes, class discussions, role plays). There are quizzes and reflective activities embedded in the cartoons to ensure student engagement, comprehension, and critical thinking. Factsheets are provided after each lesson to summarise and reinforce key content.

In line with the *OurFutures* prevention model and principles of effective tobacco and e-cigarette prevention programs [19, 20, 27], the *OurFutures Vaping Program* adopts a harm minimisation and comprehensive social influence approach [38]. This includes providing evidence-based information about e-cigarettes and tobacco smoking, normative education to correct misperceptions on use, and resistance skills training. Whilst focused on e-cigarettes, the program takes an integrated approach in addressing e-cigarette use and tobacco cigarette use. Although they are distinct behaviours with unique characteristics, simultaneously targeting both behaviours by addressing core principles that have been effective in tobacco and drug prevention is an efficient and cost-effective strategy. An overview of the *OurFutures Vaping Program* content is provided in Table 2.

**Table 2:** Overview of the *OurFutures Vaping Program* content

Lesson	Key messages
**1**	• The harmful chemicals in e-cigarettes • Prevalence and patterns of vaping/cigarette smoking • Short- and long-term consequences of vaping • Reasons young people choose to, or not to, vape
**2**	• The positive portrayal of vaping on social media • Vaping and smoking as ineffective ways to cope • Nicotine and the developing brain • Where to seek help for vaping, smoking and mental health • Assertive communication and refusal skills
**3**	• Vaping/smoking and the law • Short- and long-term consequences of smoking cigarettes • Marketing tactics and the influence of social media • The links between vaping and smoking • Help-seeking for vaping, smoking and mental health
**4**	• Assertive communication and refusal skills • Signs of nicotine dependence • Where to seek help and strategies to break the cycle of nicotine dependence • The benefits of avoiding or stopping vaping

Consultations were conducted with students and teachers throughout the design phase to construct character profiles and storylines for the cartoons and ensure the program was feasible, acceptable, and well aligned with the latest evidence and health education curricula. This included student surveys, focus groups, interviews, and input from end-users through several public webinars, school presentations and Q&A sessions. Finally, the materials underwent expert review to ensure it aligned with the latest scientific evidence. Modifications were made based on the feedback provided by these groups. This process builds on the extensive development work conducted for the previous *OurFutures* programs, which were co-designed and refined by > 210 young people and > 390 teachers, parents, and health and education experts.

#### Active control condition

Schools allocated to the control condition will implement health education as usual in their Health and Physical Education lessons. As drug education is mandatory within the Australian health education curriculum, these schools serve as an ‘active control’. A logbook will be completed by teachers at control schools to understand the amount and format of e-cigarette or tobacco cigarette education delivered to their Year 7/8 students. Control schools will be offered access to the intervention at the end of the study.

#### Assessments

All students in control and intervention groups will be invited to complete an online self-report survey in a supervised classroom setting at baseline, immediately post intervention, and 6-, 12-, 24- and 36-months post baseline to examine lasting effects (see Table 3). Students absent for follow-up surveys will be contacted by research staff (using details provided during registration) and invited to complete the survey outside of school time. Hardcopy surveys will be made available to schools/participants on request, with data entered and checked by project personnel. All data will be deidentified and linked using a unique code assigned at baseline. Upon completion, students will go into a random draw to win a $100 gift voucher, with two vouchers available per school on each assessment occasion. These strategies have been successful in previous school-based trials run by the research team, resulting in outstanding retention rates, even during the COVID-19 pandemic when there were significant disruptions to schooling (85% at 12 months and 75% at 24 months) [39].

**Table 3:** **Intervention and assessment timeline** *(F/U = follow up)*

	Baseline	OurFutures Vaping Program	Post-intervention follow-up	6-mth follow-up	12-mth follow-up	24-mth follow-up	36-mth follow-up
**Year**	2023	2023	2023	2023	2024	2025	2025
**Time**	Term 2	Term 2	Term 2	Term 4	Term 2	Term 2	Term 2
**Age**	12–14	12–14	12–14	12–14	13–15	14–16	15–17
**Intervention Group**	✓	✓	✓	✓	✓	✓	✓
**Control Group**	✓	-	✓	✓	✓	✓	✓

### Measures

#### Primary outcome

The primary outcome is change over time in self-reported e-cigarette use among the intervention group, over and above change in the control group. Specifically, uptake of e-cigarette use. To assess this, students will be asked “Have you ever used a vape, even one or two puffs?” (Yes/No). The primary endpoint will be the 12-month follow-up.

#### Secondary outcomes

*Tobacco ever use.* Assessed using a single item: “Have you ever tried smoking a cigarette, even one or two puffs?” (Yes/No). This item was based on items used in our previous trials [39] and the Standard High School Youth Risk Behaviour Survey [40].

*Frequency and quantity of e-cigarette and tobacco cigarette use.* Assessed using a series of questions based on those used in previous research [36, 39, 41] and the National Drug Strategy Household Survey [42].

*Knowledge about e-cigarettes and tobacco cigarettes.* Measured using a 15-item scale developed to reflect the content of the *OurFutures Vaping Program* intervention. The scale was derived from a larger pool of items that was piloted among adolescents to reflect items of varying degrees of difficulty. The items assess a range of topics such as the short- and long-term effects/harms of e-cigarettes and tobacco cigarettes, common myths about e-cigarettes and tobacco cigarettes, e-cigarette and tobacco cigarette laws in Australia, and the prevalence of e-cigarette and tobacco cigarette use among young people in Australia. Response options include: True/False/Don’t know.

*Motives to use e-cigarettes.* Assessed using an adapted version of the Tobacco Motives Inventory [43]. The scale comprises 15 potential motives for vaping across four subscales: Social Motives, Self-enhancement Motives, Boredom Relief Motives, and Affect Regulation Motives. Items will be rated on a 5-point Likert scale from 0 (not at all true) to 4 (very true).

*Attitudes towards e-cigarettes.* Assessed using an adapted and validated version [44] of the Smoking Expectancy Scale for Adolescents [45]. The scale includes 43 items relating to the potential consequences of smoking an e-cigarette (e.g., “feel more relaxed”, “get hooked”) across eight subscales: Health Costs, Appearance – Presentation Costs, Social Costs, Addiction, Social Benefits, Affect Control, Boredom Reduction, and Weight Control. Items are rated on a 10-point Likert scale from 0 (completely unlikely) to 9 (completely likely).

*Intentions.* Two single items based on those from our previous school-based trials [36, 39] will be used to assess intentions to use e-cigarettes and tobacco cigarettes in the next year. Responses will be rated on a 5-point scale from 0 (Certain not to try) to 4 (Certain to try).

*E-cigarette refusal skill techniques.* Assessed using items adapted from a measure developed to assess drug refusal skill techniques [46]. Participants will report their level of confidence using five refusal skills in a scenario where someone has asked them to vape. Responses will be rated on a 5-point scale from 1 (Very unconfident) to 5 (Very confident).

*Self-efficacy to resist peer pressure.* Assessed using an adapted version of the Resistive Self-Regulatory Efficacy Scale [47], with two items (those relating to the ‘use of crack’ and ‘sexual intercourse’) removed, and an item relating to vaping added. Participants rate each of the 9 items on a 7-point scale from 1 (Not well at all) to 7 (Very well). Items will be summed to generate a total score, with higher scores indicating greater self-efficacy to resist peer pressure.

*Mental health.* Depressive symptoms over the past 7-days will be assessed via the Patient Health Questionnaire-8 (PHQ-8), and 8-item scale validated for use among adolescents [48, 49]. Anxiety symptoms over the past 7-days will be assessed via the Patient Reported Outcomes Measurement Information System [50] using the 13-item anxiety scale from the pediatric item bank. The 6-item Kessler Psychological Distress Scale (K6) [51] will be used to assess symptoms of psychological distress risk for serious mental illness. The K6 has demonstrated good internal consistency in youth samples (α = 0.86) [52]. Internalising and externalising symptoms will be assessed using the 25-item Strengths and Difficulties Questionnaire [53]. Internalising symptoms are determined by summing the Emotional and Peer Problems subscales. Externalising symptoms are determined by summing the Conduct and Hyperactivity subscales. The Short Warwick–Edinburgh Mental Well-being Scale [54] will be used to assess mental wellbeing. The scale comprises 7 items and has been validated among adolescents [55]. Perceptions of Stress which will be measured by the Perceived Stress Scale (PSS), a 10-item self-report scale validated for use with adolescents [56–58].

*Health-related quality of life.* Assessed using the Child Health Utility 9D instrument (CHU9D) [59]. This instrument has been validated for self-completion by young people aged 7–17 years in Australia and can be used to derive utility weights that are, in turn, used to estimate quality-adjusted life years (QALYs) [59].

*Resource utilisation.* The utilisation of healthcare and other related services will be measured using a self-report resource use questionnaire (RUQ) that has been developed for use in previous studies [60, 61]. Participants will be asked to provide self-report data on healthcare resource use, such as: the number of contacts with primary care and specialist healthcare professionals; use of prescription medications; hospital admissions; emergency department presentations; and engagement with headspace services. Additional questions will be asked in relation to educational impacts, including school absence days. Healthcare resource use will be valued using unit prices obtained from publicly available cost schedules, such as the Medicare Benefits Schedule (MBS), Pharmaceutical Benefits Schedule (PBS) and National Hospital Cost Data Collection (NHCDC). Hourly wages will also be based on data provided by schools, where applicable, or the Australian Bureau of Statistics.

#### Additional measures

Additional measures include demographic information comprising age, sex, gender, cultural and linguistic diversity attributes, academic performance, and truancy rates. To measure socioeconomic status, students will complete the Family Affluence Scale III [62] and provide their home postcode, which will be used in addition to each school’s Index of Community Socio-Educational Advantage score. Alcohol use will be assessed using items from our previous trials [39]. Emotional regulation will be measured using the Emotion Regulation Questionnaire for Children and Adolescents [63].

#### Process evaluation

After the final lesson of the *OurFutures Vaping Program*, students and teachers in the intervention group will be asked to complete an online questionnaire to evaluate the program, and teachers will complete a logbook to assess fidelity and implementation barriers and facilitators. Using measures developed and employed in our previous school-based trials [64], the student evaluation will assess outcomes including acceptability, feasibility, engagement, and positive/negative aspects of the *OurFutures Vaping Program*. Teacher outcomes will include adherence to the intervention, student engagement and understanding, program acceptability, ease of use, educational quality, likelihood of use in the future, and any barriers or enablers to implementation. Website analytics will provide objective data on the dose and timing of intervention delivery.

### Analysis

#### Intervention effects

Data analysis will be conducted on an intention-to-treat basis, whereby all randomised students will be analysed in the groups that they were originally assigned. Generalized mixed effects regression will investigate whether receiving the intervention reduces the likelihood of primary and secondary outcomes (e.g., logistic regression for dichotomous outcomes, poisson regression for count outcomes, linear regression for continuous outcomes). Analyses will be conducted in R, using the *lme4* package [65]. To account for within-person and within-school dependency in the data, models will include participant and school as nested random intercepts; and participant and time as random slopes. We will also test different specifications of time (linear, quadradic & categorical) to determine the best fitting model for the data. Model fit will be compared using likelihood ratio tests, AIC and BIC statistics. The effect of greatest interest will be the time × group interaction for the primary outcome, which reflects the relative average 12-month change in the log odds of the outcome for the intervention group compared to control, adjusting for baseline differences. Due to loss to follow-up, we reasonably expect some outcome data to be missing. Mixed-effects models use maximum likelihood estimation (MLE), producing unbiased estimates when data is assumed to be not missing completely at random. Missing data will be explored by examining baseline differences on the outcome and other potential confounding variables between those retained and those lost to follow-up. Sensitivity analysis will examine the impact of potential covariates related to missingness by including those predictors in the imputation model during multiple imputation.

#### Cost-effectiveness

The base case analysis will be undertaken using a partial societal perspective, alongside an additional analysis from a health sector perspective. Area-under-the-curve methods will be applied to CHUD9D data to estimate the QALYs associated with the *OurFutures Vaping Program* and active control condition. The costs that accrue across each trial arm will be calculated as the sum of: all relevant intervention delivery costs; and the cost of utilising additional healthcare and other related services. The cost of delivering the *OurFutures Vaping Program* and health education as usual will be estimated based on a detailed accounting of resources required to deliver each respective program. This will consider all costs associated with intervention delivery and usual education, including staff and teacher time alongside program materials. Data from the RUQ will be used to estimate the cost of utilising additional healthcare and other related services. The cost-effectiveness of the *OurFutures Vaping Program* – when compared to health education as usual – will be evaluated using the incremental cost-effectiveness ratio (ICER) as the main measure of cost-effectiveness. That is, the difference in mean costs divided by the difference in mean QALYs between the intervention and control arms.

A cost-consequences analysis will also be done to produce a dashboard of all relevant costs and outcomes resulting in each trial arm. Additional cost-effectiveness ratios can then be estimated by adopting primary and secondary outcomes as the denominator (e.g., the cost per student not engaging in vaping behaviours and the cost per unit gained on the PHQ-8). Standardised economic evaluation techniques, such as Fieller’s theorem and non-parametric bootstrapping, will be used to estimate confidence intervals around each cost-effectiveness ratio. Economic modelling can also be undertaken, if applicable, to estimate the long-term costs and outcomes that may accrue beyond the timeframe of the cluster RCT and to estimate the cost-effectiveness and/or budget impact of scaling up the intervention across Australian schools.

### Data Safety and Monitoring

A data monitoring committee consisting of researchers independent to the core trial team will meet quarterly to review trial management, progress, and oversee statistical analysis. The committee will monitor recruitment, dropouts, and any adverse events in consultation with the lead researchers in each state. All adverse events will be recorded, and serious adverse events will be immediately reported to the governing Human Research Ethics Committee/s.

## Discussion

This paper describes the study design and protocol of the *OurFutures Vaping Trial;* the first evaluation of a school-based eHealth preventive intervention targeting e-cigarette use among young Australians. By building on effective principles of tobacco and e-cigarette prevention [27], and the successful *OurFutures* prevention model which harnesses innovative eHealth technology, this novel evidence-based program offers a highly feasible and rapidly scalable solution to a global public health priority. The simultaneous targeting of both e-cigarettes and tobacco cigarettes is another key strength of the program. However, given the study will span three Australian states and is anticipated to include > 3,300 secondary school students, it will rely on self-report measures which may be subject to reporting biases. Nevertheless, the study has the potential to improve the immediate health and wellbeing of young people, while also safeguarding them from serious health impacts and addiction caused be e-cigarettes and tobacco cigarettes. If effective, the intervention will be readily accessible via the publicly available OurFutures platform and has the potential to make substantial health and economic impact. Without such intervention, young Australians will be the first generation to use nicotine *at higher rates* than previous generations, thereby undoing decades of effective tobacco control.

## Electronic supplementary material

Below is the link to the electronic supplementary material.

Supplementary Material 1

## Data Availability

Upon completion of the study, data will be made available upon reasonable request to the study team.

## Abbreviations

CHU9D: Child Health Utility 9D instrument
CONSORT: Consolidated Standards of Reporting Trials
EVALI: e-cigarette or vaping use-associated lung injury
ICER: incremental cost-effectiveness ratio
K6: 6-item Kessler Psychological Distress Scale
NSW: New South Wales
MBS: Medicare Benefits Schedule
MLE: maximum likelihood estimation
NHCDC: National Hospital Cost Data Collection
OR: Odds Ratio
PBS: Pharmaceutical Benefits Schedule
PHQ-8: Patient Health Questionnaire-8
QALYS: quality-adjusted life years
QLD: Queensland
RCT: Randomised controlled trial
RUQ: resource use questionnaire
SPIRIT: Standard Protocol Items:Recommendations for Interventional Trials
US: United States
WA: Western Australia
